# Differences in Survival Associated With the First Antiseizure Medication in People With Dementia and Epilepsy

**DOI:** 10.1212/WNL.0000000000214929

**Published:** 2026-04-17

**Authors:** Johan Zelano, David Larsson, André Idegård, Francesco Brigo

**Affiliations:** 1 Department of Clinical Neuroscience, Institute of Neuroscience and Physiology, the Sahlgrenska Academy at the University of Gothenburg, Sweden;; 2 Department of Neurology, Sahlgrenska University Hospital, member of ERN Epicare, Gothenburg, Sweden;; 3 Wallenberg Center of Molecular and Translational Medicine, Gothenburg University, Sweden; and; 4 Innovation, Research and Teaching Service, Teaching Hospital of the Paracelsus Medical Private University, Azienda Sanitaria dell'Alto Adige, Bolzano-Bozen, Italy.

## Abstract

**Background and Objective:**

Epilepsy is an increasingly recognized comorbidity in neurocognitive disorders. Although experts recommend newer antiseizure medications (ASMs), whether there are real-world benefits of selecting particular ASMs for epilepsy in dementia remains unknown. Our objective was to examine whether different ASMs are associated with differences in survival in persons with epilepsy and dementia.

**Methods:**

A cohort study was conducted using Swedish national registers. We included individuals with dementia who received ASM treatment after a diagnosis of epilepsy after January 1, 2006. Data were analyzed until December 2023. Dispensed ASMs (Anatomical Therapeutic Code N03) were used to identify treatment; patients were categorized as starting one of the 4 most common ASMs (carbamazepine, levetiracetam, lamotrigine, valproate) or other ASMs. Recurrent prescriptions were used to determine treatment duration. Associations between all-cause death and ASMs were assessed by Cox proportional hazards regression. We also assessed causes of death and the risk of cardiovascular death.

**Results:**

In Sweden, between 2006 and 2023, we included 5,764 individuals (2,811 men [48.8%] and 2,953 women [51.2%]) using their first ASM after a diagnosis of epilepsy and dementia. Carbamazepine (n = 1,578) was the most common ASM before 2015 and levetiracetam (n = 2098) most common thereafter. In Kaplan-Meier analyses and Cox regression models adjusting for age, sex, year of ASM start, and comorbidities, valproate (n = 746) was associated with increased adjusted hazard ratio (aHR) of death (1.34, 95% CI 1.20–1.48), in contrast to lamotrigine (n = 922, aHR: 0.84, 95% CI 0.75–0.93) and levetiracetam (n = 2098, aHR: 0.93, 95% CI 0.85–1.03). The risks were similar in analyses using restricted mean survival time, propensity score–matched sets of participants, balancing weights in regression models, and cases with epilepsy onset in existing dementia. Cardiovascular causes of death were more common among users of valproate or carbamazepine than among users of lamotrigine and levetiracetam. Compared with carbamazepine, valproate was associated with increased risk of cardiovascular death (aHR: 1.30; 95% CI 1.11–1.52) while lamotrigine (aHR 0.79; 95% CI 0.66–0.94) was associated with a reduced risk.

**Discussion:**

In this population-wide cohort study, use of valproate was associated with the highest risk of death in persons with epilepsy and dementia. Lamotrigine and, in some models, levetiracetam were associated with better survival than both valproate and carbamazepine. This provides real-world support for existing expert guidelines.

## Introduction

Epilepsy affects at least 5% of individuals with dementia, and the International League Against Epilepsy has highlighted substantial gaps in knowledge regarding seizure management in this vulnerable population.^1,2^ Current European treatment guidelines recommend lamotrigine or levetiracetam, while raising concerns about older antiseizure medications (ASMs) such as valproate.^3^ These recommendations are primarily based on expert opinions and short-term tolerability studies in older patients.^4,5^ Emerging evidence also points to associations between vascular risk factors, neurodegeneration, and late-onset epilepsy, suggesting that ASMs with lower impact on cardiovascular risk may be more suitable for patients with cognitive decline.^6,7^ Despite these concerns, older ASMs including valproate continue to be widely prescribed for elderly individuals and those with dementia.^8-11^

We recently investigated mortality in persons with epilepsy and found that dementia significantly increased the risk of death.^12^ However, it remains unclear whether the choice of ASM influences survival in patients with both dementia and epilepsy. Traditional studies face several limitations, including fragmented follow-up outside neurology departments, the extended observation period required to assess mortality, and challenges in obtaining informed consent from cognitively impaired individuals. To overcome these barriers, we used nationwide registry data to examine whether survival outcomes varied according to the initial ASM prescribed. We hypothesized that the expert-recommended ASMs—lamotrigine and levetiracetam—would be associated with better survival compared with older alternatives. To test this, we conducted a longitudinal cohort study based on Swedish registry data, including all individuals on their first ASM treatment after a diagnosis of epilepsy and dementia.

## Methods

### Registers

The study used cross-referenced data from 3 Swedish national registers: the National Patient Register, the Cause of Death Register, and the Prescribed Drug Register. The National Patient Register includes diagnostic codes and information on all inpatient and specialized outpatient care in Sweden, with outpatient data available from 2005 and inpatient data from 1987 onward. The Prescribed Drug Register contains records of all dispensed prescriptions in Sweden since July 1, 2005. The National Board of Health and Welfare identified all individuals with a diagnosis of epilepsy after January 1, 2006. We accessed register data up to the end of 2023. Before analysis, all data were cross-referenced and anonymized by the respective register authorities.

### Study Cohort

The National Board of Health and Welfare identified all patients with a diagnosis of epilepsy (G40) as an inpatient or outpatient diagnosis after 2005. In this data set, we identified 6,205 individuals with diagnostic codes for both epilepsy and dementia who received an ASM prescription after January 1, 2006 (after the first epilepsy diagnosis). Dementia was defined by the presence of International Classification of Diseases, 10th Revision (ICD-10) codes F00–F03, G30, or G31; epilepsy by code G40; and ASM exposure by a record in the Prescribed Drug Register indicating dispensation of a prescription with Anatomical Therapeutic Chemical (ATC) code N03. We categorized patients receiving the 4 most common ASMs and the rest as “others.” To ensure accurate temporal alignment, we excluded 793 individuals who had already discontinued their first ASM before their diagnosis of epilepsy, indicating previous exposure. For exposure, we only considered first ASMs prescribed after a diagnosis of epilepsy.

### Variables

Data on age and sex (binary in the registers, refers to male/female legal sex) were obtained from the National Patient Register. The initial ASM was identified using ATC codes in the Prescribed Drug Register. Comorbidities were determined based on ICD-10 diagnostic codes, and cause of death was retrieved from the Cause of Death Register, categorized according to the ICD-10 chapter of the underlying cause. Specific comorbidities were defined as follows: stroke (I60–I64), traumatic brain injury (TBI) (S06, S020, S021 S027, S029), brain tumor (C71, C793, D430, D32, D330), brain infection (A84, B004, A390, G00, G01, A17, A066, B431, G060, G079, A85, A86, B011, B020, B050, B262, B602, A87, B003, B010, B051, B261, B021, B375, B384, B582, G02), diabetes (E10, E11), cardiovascular diseases (I00–I99, excluding I10, I60-64, and I11.9-I16), mood or anxiety disorders (F3, F4), intellectual disability (F70-79), congenital disorder (Q00-Q07, Q90-Q99), and cancer (C codes). Antipsychotics were identified as an indicator of potential behavioral symptoms by prescription for any ATC code N05A and entered as a time-updated variable (first occurrence). As epilepsy severity indicators, we extracted hospitalizations with status epilepticus (SE) or epilepsy as main diagnosis, identified by ICD-10 codes G41 and G40, respectively. The Charlson Comorbidity Index (CCI) was calculated using a validated algorithm previously applied to Swedish registry data.^13^ Dementia subtypes were extracted based on ICD codes used in a recent validation study and categorized by the latest specific code: Alzheimer dementia (F00, G30), vascular dementia (F01), dementia with Lewy bodies (F028), Parkinson disease dementia (F023), and frontotemporal dementia (F020, G310).^14^

### Statistical Analysis

Descriptive statistics were reported as proportions with corresponding 95% CIs. Survival was conducted using Kaplan-Meier estimates, with time zero defined as the date of the first ASM prescription after an epilepsy diagnosis. Patients were censored at death, on December 31, 2023, or at discontinuation of their initial ASM. Cox proportional hazards regression was used to adjust for age (continuous), year of ASM start (continuous), sex, and comorbidities (all categorical at baseline, except antipsychotics, which was time-updated). Given the difference in early mortality and visual inspection of Kaplan-Meier curves suggesting that the proportional hazard assumption may not hold beyond 5 years between all ASMs and carbamazepine, which was used as reference, we also performed analyses truncated at this time point. In addition, we performed restricted mean survival time (RMST) analyses until 5 years and over the entire study period. Adjusted differences in RMST were modeled using pseudo-observations and linear regression, adjusting for the age, year of ASM start, sex, and comorbidities. ASM therapy was determined based on prescription intervals: renewal within 12 months indicated continued treatment, whereas discontinuation was defined as no prescription within 3 months after the last recorded dispense. In additional Cox regression analyses, we adjusted the models for CCI as opposed to individual comorbidities, epilepsy severity markers (time-updated first occurrence of status epilepticus or hospitalization with epilepsy as a main diagnosis), or dementia subtypes.

In addition to standard Cox regression, we attempted to adjust for differences in baseline characteristics through 2 strategies: matching and balancing. Propensity score–matched analyses comparing carbamazepine (reference) with the other ASMs was performed using the R package *Matchit,* based on propensity scores derived from age, sex, comorbidities at baseline (not antipsychotics), CCI, and year of treatment initiation. This resulted in matched cohorts of 1,578 levetiracetam users, 922 lamotrigine users, and 746 valproate users, each matched to an equal number of carbamazepine users. The matching was “nearest neighbour” with standard setting (no replacement and no caliper). The matching was assessed using AMD plots and could not fully compensate for differences in covariates. We also performed weighted analyses using entropy balancing, implemented through the R package *Weightit*, to generate weights that exactly balanced covariate means. Variables used for weighting were year, age, CCI, comorbidities at baseline (not antipsychotics), and sex. In addition, we modeled the risk of cardiovascular death using Cox regression, both with and without entropy weights for covariate balance.

In sensitivity analyses, we analyzed follow-up until 7 years (rather than 5 years) and restricted inclusion to those starting their first ASM before 2021 to ensure at least 2 years of follow-up.

Analyses were conducted using SPSS version 29.0 and R version 4.5.0 (employing the *MatchIt_4.7.2*, *survival_3.8-3*, *cobalt_4.6.1*, *Pseudo1.4.3*, *survRM2_1.0-4* and *WeightIt_1.5.1* packages). The University of Gothenburg Microsoft Copilot (AI) service was used for debugging/improving R-code.

### Standard Protocol Approvals, Registrations, and Patient Consents

The study was approved by the Swedish Ethical Review Authority, under approval number 2023-5598. Because of the register-based nature of the study, the need for consent was waived.

### Data Availability

The underlying data are available from Swedish national registers on request but cannot be shared by the authors because of confidential agreements with the register holders and Swedish privacy regulations. Related documents will be shared on reasonable request.

## Results

**Figure 1:**
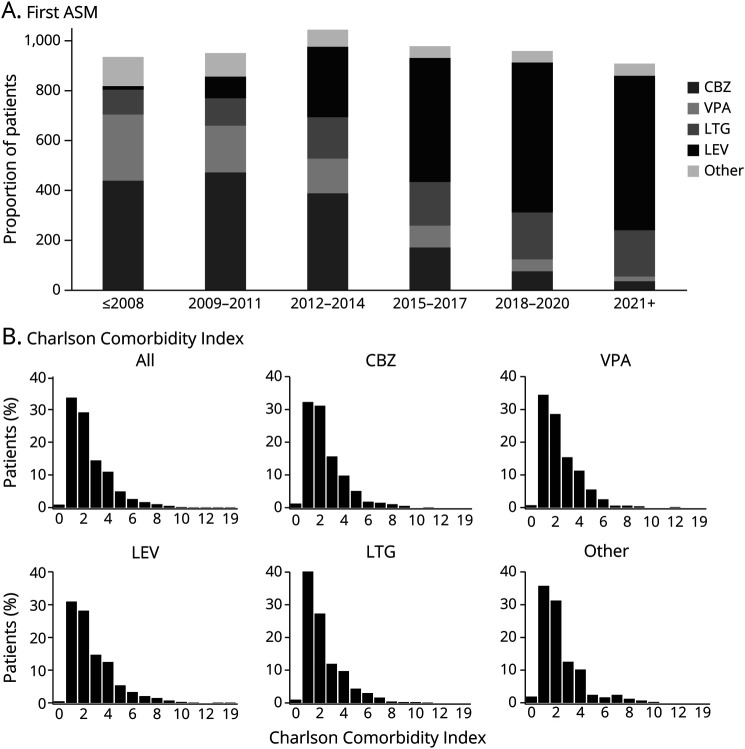
Proportions of the First Antiseizure Medication Throughout the Study Period (A) and Charlson Comorbidity Index of Users of the Different ASMs (B) ASM = antiseizure medication; CBZ = carbamazepine, VPA = valproate, LTG = lamotrigine, LEV = levetiracetam.

The final cohort included 5,764 patients with ASM treatment after a diagnosis of epilepsy and dementia. Of the entire cohort, 49% were male and 51% female (Table 1). More than 86% were older than 65 years at inclusion. Cardiovascular disease, stroke, and antipsychotic use were the most common comorbidity variables. In patients younger than 40 years at inclusion, intellectual disability (38%, 95% CI 28–48, *p* < 0.001) and inborn disorders (17%, 95% CI 10–26, *p* < 0.001) were significantly more common than in individuals aged older than 65. Prescription patterns shifted over time, with carbamazepine being the most commonly initiated ASM before 2015 and levetiracetam becoming the most common choice thereafter (Figure 1A). Valproate was the second most frequently prescribed ASM before 2008, but its use declined in subsequent years. Analysis of the CCI revealed that lamotrigine users had a lower overall comorbidity burden compared with those treated with other ASMs (Figure 1B). Patients initiating lamotrigine or levetiracetam were more often female than those starting carbamazepine, and lamotrigine users were less likely to have cardiovascular disease than patients starting other ASMs (Table 1).

**Table 1:** Cohort Demographics, Comorbidities, Dementia Types, and Severity Indicators

	Total (n = 5,764)		CBZ (n = 1,578)		VPA (n = 746)		LTG (n = 922)		LEV (n = 2098)		Other (n = 420)	
	n	%	n	%	n	%	n	%	n	%	n	%
Male	2,811	48.8	828	52.5	367	49.2	427	46.3	984	46.9	205	48.8
Female	2,953	51.2	750	47.5	379	50.8	495	53.7	1,114	53.1	215	51.2
Age												
<40	93	1.6	15	1.0	12	1.6	16	1.7	24	1.1	26	6.2
40–65	671	11.6	233	14.8	82	11.0	111	12.0	199	9.5	46	11.0
>65	5,000	86.7	1,330	84.3	652	87.4	795	86.2	1875	89.4	348	82.9
Comorbidities												
Cancer	592	10.3	166	10.5	73	9.8	80	8.7	237	11.3	40	9.5
Stroke	2058	35.7	592	37.5	252	33.8	275	29.8	813	38.8	126	30.0
TBI	443	7.7	126	8.0	54	7.2	57	6.2	181	8.6	25	6.0
Brain tumor	164	2.9	43	2.7	10	1.3	26	2.8	75	3.6	10	2.4
Brain infection	67	1.2	20	1.3	10	1.3	5	0.5	26	1.2	6	1.4
Diabetes	982	17.1	250	15.8	129	17.3	155	16.8	385	18.4	63	15.0
Cardiovascular	3,325	57.7	913	57.9	434	58.2	478	51.8	1,280	61.0	220	52.4
Mood or anxiety disorder	575	10.0	158	10.0	86	11.5	89	9.6	200	9.5	42	9.9
Intellectual disability	106	1.8	26	1.6	10	1.3	20	2.2	33	1.6	17	4.0
Inborn disorder	198	3.4	46	2.9	22	2.9	38	4.1	79	3.8	13	3.1
Antipsychotics	2,295	39.8	629	39.9	300	40.2	389	42.2	782	37.3	195	46.4
Specific dementia												
Alzheimer	1989	34.5	508	32.2	243	32.6	357	38.7	760	36.2	121	28.8
VAD	1,520	26.4	442	28.0	196	26.3	216	23.4	561	26.7	105	25.0
PDD	78	1.4	12	0.8	8	1.1	14	1.5	36	1.7	8	1.9
DLB	125	2.2	27	1.7	14	1.9	25	2.7	44	2.1	15	3.6
FTD	124	2.2	32	2.0	16	2.1	26	2.8	41	2.0	9	2.1
Epilepsy												
Before dementia	283	4.9	87	5.5	52	7.0	44	4.8	67	3.2	33	7.9
Admission SE as main diagnosis	238	4.1	52	3.3	26	3.5	21	2.3	107	5.1	32	7.6
Admission Ep	3,189	55.3	829	52.5	446	59.8	513	55.6	1,210	57.7	191	45.5

Abbreviations: CBZ = carbamazepine; DLB = dementia with Lewy bodies; Ep = epilepsy as main diagnosis; FTD = frontotemporal dementia; LEV = levetiracetam; LTG = lamotrigine; PDD = Parkinson disease dementia; Preexisting = epilepsy before first dementia; SE = status epilepticus; VAD = vascular dementia; VPA = valproate.

### Survival

**Figure 2:**
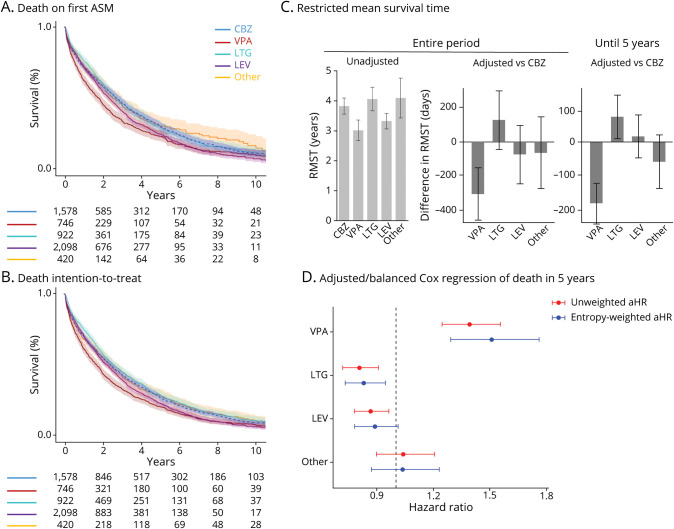
(A) Kaplan-Meier Curve of Survival After Starting a Particular First ASM With Censoring at Discontinuation (B) Kaplan-Meier curve of survival after starting a particular first ASM (intention to treat). (C) Restricted mean survival time analyses, both crude survival times in unadjusted analysis and the difference to CBZ in adjusted analyses. (D) Unbalanced and entropy-balanced Cox regression on the hazard of death in the first 5 years, adjusted and balanced for both individual comorbidities and CCI. ASM = antiseizure medication; CBZ = carbamazepine, VPA = valproate, LTG = lamotrigine, LEV = levetiracetam.

In Kaplan-Maier analysis, patients using valproate had a higher mortality rate during the first 5 years of follow-up (Figure 2A). This pattern remained consistent even if the analysis was conducted in an intention-to-treat manner, with no censoring at first ASM discontinuation (Figure 2B). We also performed RMST analyses (Figure 2C). When analyzed up to 5 years, valproate was the only ASM with significantly lower RMST than carbamazepine, 768 days (95% CI 716–820) vs 950 days (95% CI 812–989), respectively. Over the entire study period, the RMST for carbamazepine was 1,396 (95% CI 1299–1,494), significantly longer than for valproate (1,101; 95% CI 976–1,227). There was no significant difference between carbamazepine and levetiracetam (1,213, 95% CI 1118–1,308), lamotrigine (1,482, 95% CI 1339–1,626), or other ASMs (1,494, 95% CI 1252–1737). In adjusted analyses, the RMST of valproate was significantly different compared with carbamazepine over the entire period (−300 days; 95% CI −452 to −148), *p* < 0.001). In an adjusted analysis up to 5 years, the RMST of valproate was significantly shorter than that of carbamazepine (−189 days; 95% CI −253 to −126), *p* < 0.001) and the RMST of lamotrigine was significantly longer than that of carbamazepine (75 days; 95% CI 8–142, *p* = 0.03).

### Cox Regression

Hazard of death was assessed using Cox regression models. The main models were adjusted for age, sex, and year of ASM initiation, followed by additional adjustments for individual comorbidities defined by ICD codes and neuroleptic use. Valproate was associated with an increased hazard of death over the entire study period, while lamotrigine was associated with a lower hazard (Table 2). Valproate users also had a significantly higher risk of death within the first 5 years (adjusted hazard ratio [aHR] 1.39; 95% CI 1.24–1.55), whereas lamotrigine was associated with a lower risk (aHR 0.88; 95% CI 0.78–0.98), compared with carbamazepine.

**Table 2:** Cox Regression

	aHR (95% CI)	
Death		
CBZ	ref	
VPA	1.34 (1.20–1.48)	<0.001
LTG	0.84 (0.75–0.93)	0.001
LEV	0.93 (0.85–1.03)	0.177
OTHER	1.02 (0.89–1.17)	0.786
Death first 5 y		
CBZ	ref	
VPA	1.39 (1.24–1.55)	<0.001
LTG	0.81 (0.72–0.91)	<0.001
LEV	0.89 (0.80–0.99)	0.036
OTHER	1.03 (0.89–1.20)	0.663

Abbreviations: CBZ = carbamazepine; LEV = levetiracetam; LTG = lamotrigine; VPA = valproate.

Risk of death while on the first antiseizure medication. Adjusted for age, sex, year of ASM start, and comorbidities.

We also performed intention-to-treat analysis, based on the first started ASM (eTable 1). The results were similar to those of the main analysis, but the HRs were slightly lower. Additional analyses adjusting for CCI rather than individual comorbidities (eTable 2), dementia subtypes (eTable 3), and epilepsy severity indicators (hospitalization for status epilepticus and epilepsy, eTable 4) showed similar results. Stratified analysis by sex (eTable 5) revealed no significant differences: among male patients, the aHR for valproate vs carbamazepine was 1.38 (95% CI 1.19–1.61; model 2), and among female patients, the aHR was 1.31 (95% CI 1.12–1.52). In several, but not all, analyses of 5-year death, levetiracetam also showed a significantly lower hazard of death compared with carbamazepine (main analysis aHR 0.89; 95% CI 0.80–0.99).

### Propensity Score–Matched and Balanced Analyses

In an alternative strategy to address confounding, we compared propensity score–matched analyses to compare carbamazepine with other ASMs in matched subsets of the cohort. We matched cases of the investigated ASM to the closest matches among carbamazepine users, using all available information (comorbidities plus CCI); the best matching was achieved for valproate vs carbamazepine (eFigure 1). In the matched data sets, valproate was associated with an increased hazard of death (aHR 1.33; 95% CI 1.17–1.51), while lamotrigine was associated with a lower hazard (aHR 0.88; 95% CI 0.78–1.00) compared with carbamazepine. Levetiracetam had a confidence interval exceeding 1 (aHR 0.93; 95% CI 0.80–1.09). Because this method could not achieve good matches for lamotrigine and levetiracetam, we performed balanced analyses by applying weights based on comorbidities and CCI, age, sex, and year of treatment initiation to all cases in the data set. Covariate balance achieved through entropy weighting was superior to that obtained using propensity scores (eFigure 2). In the entropy-balanced analysis (Figure 2B), the aHR for death within the first 5 years was 1.51 (95% CI 1.29–1.76) for valproate, 0.83 (95% CI 0.73–0.94) for lamotrigine, and 0.88 (95% CI 0.77–1.01) for levetiracetam.

### Causes of Death

**Figure 3:**
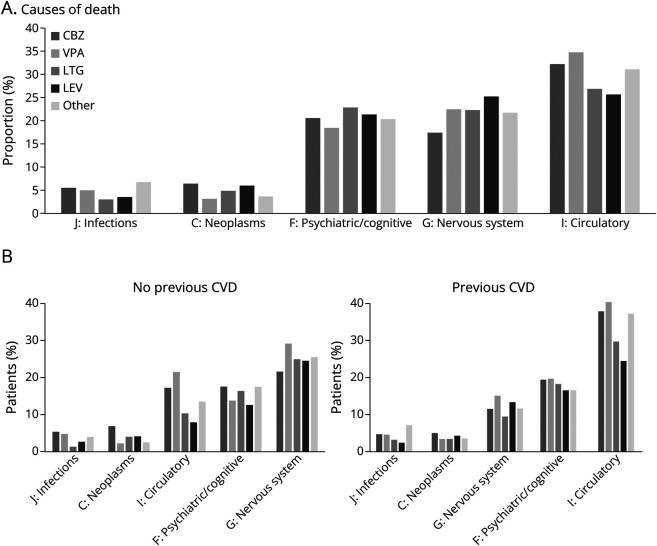
(A) Causes of Death Among Diseased in the Cohort by ICD-10 Chapter (B) Causes of death in patients with or without preexisting cardiovascular disease (CVD). CBZ = carbamazepine, VPA = valproate, LTG = lamotrigine, LEV = levetiracetam.

Causes of death were classified according to the ICD-10 chapter of the underlying cause. The most frequent categories included vascular diseases, nervous system diseases, psychiatric and behavioral disorders (including cognitive), neoplasms, and infections. When comparing proportion of deaths across first-line ASM groups (Figure 3A), vascular causes were significantly more common (*p* < 0.001 in pairwise comparisons of proportions) among users of carbamazepine (29%, 95% CI 27–32) or valproate (33%, 95% CI 29–36) than among those prescribed lamotrigine (20%, 95% CI 18–23) or levetiracetam (18%, 95% CI 16–20). Stratification by preexisting cardiovascular disease revealed that among patients without such conditions, psychiatric/cognitive and nervous system disorders were more common causes of death than cardiovascular disease (figure 3B). However, even within this subgroup, users of carbamazepine or valproate were more likely (*p* < 0.001 for pairwise comparisons of proportions) to have a cardiovascular cause of death (carbamazepine 17%, 95% CI 15–20; valproate 22%, 95% CI 17–26) compared with users of lamotrigine (10%, 95% CI 8–13) or levetiracetam (8%, 95% CI 6–10). Cox regression models stratified by preexisting cardiovascular disease showed that valproate was associated with an increased hazard of death in both groups. Among patients without preexisting cardiovascular disease, the aHR for death of any cause on valproate was 1.34 (95% CI 1.14–1.59), and among those with cardiovascular disease, the aHR for death of any cause on valproate was 1.34 (95% CI 1.17–1.53). We further analyzed the specific risk of cardiovascular death using Cox regression censored at discontinuation of the first ASM and adjusted for age, sex, year of ASM initiation, and comorbidities. Compared with carbamazepine, valproate was associated with a significantly increased risk of cardiovascular death (aHR 1.30; 95% CI 1.11–1.52), while lamotrigine (aHR 0.79; 95% CI 0.66–0.94) was associated with a reduced risk. This was not the case for levetiracetam (aHR 0.86, 95% CI 0.73–1.02, *p* = 0.08). These associations were more pronounced when analyses were limited to the first 5 years of follow-up; aHR of cardiovascular death for valproate was 1.41 (95% CI 1.19–1.66), for lamotrigine 0.78 (95% CI 0.65–0.95), and for levetiracetam 0.84 (0.70–1.00, *p* = 0.047). Balanced Cox regression with entropy weights confirmed the elevated risk of cardiovascular death associated with valproate compared with carbamazepine (eFigure 3).

### Sensitivity Analyses

Multiple sensitivity analyses were performed to assess the robustness of the findings. Truncating follow-up at 7 years did not materially affect the results (eTable 6). In addition, restricting the cohort to individuals included before 2021 (to ensure a minimum of 2 years of follow-up) yielded consistent outcomes, with no significant differences observed in the findings (eTable 7). To isolate epilepsy starting in existing dementia, we restricted one analysis to those with a first epilepsy diagnosis after 2007 and no previous dementia (ensuring epilepsy that is incident in dementia) who had not started their first ASM more than a year before the epilepsy diagnosis (a reasonable time for ASMs started in emergency departments after seizures before a neurology visit) and found similar results (eTable 8).

## Discussion

In this nationwide cohort of patients with epilepsy, dementia, and ASM treatment in Sweden from 2006 to 2023, we observed significant differences in mortality risk associated with the choice of first-line ASM. It is important to note that valproate was consistently associated with an increased hazard of death. Valproate remains a popular ASM in older patients in many countries^8,10,11,15^; however, our findings, although observational, suggest that its use warrants caution. The observation is particularly important, given the recent reports of increased use of valproate in older patients with dementia and behavioral disorders.^9^ Conversely, lamotrigine and, in some models, levetiracetam, were associated with a reduced hazard of death compared with carbamazepine. These findings align with a recent treatment guideline favoring these ASMs for persons with dementia.^3^

The mechanisms underlying the increased mortality risk with older ASMs remain unclear. Vascular causes were the most frequent underlying cause of death in our cohort and were disproportionately represented among users of carbamazepine and valproate. Both drugs can have negative effects on vascular risk factors such as serum lipids.^16,17^ However, the association is more complex for valproate, which has previously been linked to a lower risk of cardiovascular events in the general epilepsy population.^18^ Age-related variation in vascular risk may partly explain this discrepancy. In addition, the autopsy frequency in Sweden is low and diagnostic uncertainty in older patients may limit the accuracy of cause-of-death classifications. Introducing epilepsy severity variables did not alter the HR of death for any of the studies on ASMs, in line with the cause-of-death analyses where nervous system disorders were not among the top causes of death.

Among patients without preexisting cardiovascular disease, deaths attributed to psychiatric and neurologic disorders—including dementia—were more common. Use of ASMs and, in particular, valproate has been associated with cognitive decline, encephalopathy, and increased dementia risk.^5,19,20^ Although the connection to neurodegeneration remains debated,^21^ we cannot exclude the possibility that nonvascular mechanisms may also have contributed to the increased hazard of death associated with valproate. Causes of deaths on death certificates need to be interpreted with caution; preexisting dementia and absence of vascular disease may suggest neurodegeneration as the stated cause of death. By contrast, studies have reported improved cognitive outcomes in patients with Alzheimer disease and epilepsy treated with levetiracetam.^22,23^

Our study has several limitations. Over the course of the study period, several trends likely contributed to shifts in first-line antiseizure medications. Clinical guidelines increasingly favored newer ASMs such as lamotrigine and levetiracetam because of their improved tolerability, reduced drug-drug interactions, and more favorable safety profiles in older adults. At the same time, growing awareness of adverse effects associated with older ASMs—particularly valproate and carbamazepine—may have led clinicians to reconsider their use, especially in patients with cognitive impairment. Changes in prescriber training and preferences, as well as regulatory updates and market availability, also played a role. For example, newer clinicians may be more familiar with lamotrigine and levetiracetam, while warnings about valproate's teratogenicity and cognitive risks have become more prominent. In addition, the increasing focus on preserving cognitive function in aging populations has likely influenced treatment decisions, with accumulating evidence and emerging literature supporting the use of ASMs that pose less risk to neurocognitive health. These evolving factors help contextualize the observed transition in prescribing patterns over time, but differences in baseline characteristics and comorbidity burden across ASM groups introduce potential residual confounding. Patients with lamotrigine were less likely to have cardiovascular disease, perhaps due to warnings about arrhythmogenicity discussed during the study period.^24^ Lamotrigine and levetiracetam were most frequently prescribed later in the study period, resulting in shorter follow-up and fewer observed deaths. However, this limitation does not apply to valproate, which was more commonly used earlier, similar to carbamazepine. We addressed these concerns through adjustments and multiple sensitivity analyses, including time truncation and matched and weighted models, all of which yielded consistent results. Nonetheless, replication in larger data sets and, ideally, in other countries is warranted. We were also not able to study dosing of valproate, and it would be very interesting if future studies could attempt to study survival in relation to mortality in patients with dementia or older patients in general. Our cohort consisted mainly of older patients with epilepsy after dementia, so future investigations should assess whether these observations are also valid in younger individuals. The registers in Sweden do not contain information on ethnicity or sex and the generalizability regarding other race/ethnicity/nonbinary populations may be limited.

As a registry-based study, it also relies on the accuracy of the underlying data, that is, the validity of ICD-10 codes in the registers. However, while there is some variation in quality, the overall validity is high for ICD-10 codes in the NPR, including G40 to define epilepsy.^25,26^ A related issue is that it is difficult to isolate epilepsy caused by dementia in register data. We tried by restricting one sensitivity analysis to only patients with epilepsy onset after a registered code for dementia. This analysis should be interpreted with some caution; the approach probably excluded some patients with dementia-associated epilepsy because the sensitivity for dementia is considerably lower than for epilepsy in register data. The accuracy of the codes once detected is generally good. A diagnosis of epilepsy has a positive predictive value of >90% in the NPR, and in combination with a prescription for an ASM, this is likely even higher.^26,27^ A code for dementia has a positive predictive value of 96%, but for subtypes, the positive predictive values are lower: AD (64%), VaD (72%), DLB (63%), PDD (80%), and FTD (65%).^14^ We had no information on dementia severity apart from antipsychotic use. There may also be some miscoding errors. For instance, 1.6% of the population were younger than 40 at onset of their epilepsy. A proportion of these may represent true early-onset dementias, but it is also possible that cognitive symptoms in intellectual disability were miscoded as dementia. The main analyses were based on the more robust variables, so marginal miscoding errors should not influence the conclusions.

This nationwide study using comprehensive national registers, enabling long-term, population-wide follow-up, highlights significant differences in mortality risk associated with ASMs in patients with epilepsy and dementia. Valproate was consistently linked to increased mortality, underscoring the need for caution in its use among older adults with cognitive impairment. By contrast, lamotrigine and levetiracetam were associated with lower hazards of death, supporting current guideline recommendations. These findings emphasize the importance of informed ASM selection in this vulnerable population and demonstrate the value of large-scale registry data in guiding safer therapeutic strategies. Further research is needed to evaluate newer ASMs and expand treatment options for epilepsy in dementia.

## Glossary

aHR: adjusted hazard ratio
ASM: antiseizure medication
ATC: Anatomical Therapeutic Chemical
ICD-10: International Classification of Diseases, 10th Revision
RMST: restricted mean survival time

## References

[1] Subota A, Pham T, Jette N, Sauro K, Lorenzetti D, Holroyd-Leduc J. The association between dementia and epilepsy: a systematic review and meta-analysis. Epilepsia. 2017;58(6):962-972. doi:10.1111/epi.13744 28397967

[2] Beghi E, Giussani G, Costa C, et al. The epidemiology of epilepsy in older adults: a narrative review by the ILAE Task Force on Epilepsy in the Elderly. Epilepsia. 2023;64(3):586-601. doi:10.1111/epi.17494 36625133

[3] Frederiksen KS, Cooper C, Frisoni GB, et al. A European Academy of Neurology guideline on medical management issues in dementia. Eur J Neurol. 2020;27(10):1805-1820. doi:10.1111/ene.14412 32713125 PMC7540303

[4] Werhahn KJ, Trinka E, Dobesberger J, et al. A randomized, double-blind comparison of antiepileptic drug treatment in the elderly with new-onset focal epilepsy. Epilepsia. 2015;56(3):450-459. doi:10.1111/epi.12926 25684224

[5] Beyenburg S, Back C, Diederich N, Lewis M, Reuber M. Is valproate encephalopathy under-recognised in older people? A case series. Age Ageing. 2007;36(3):344-346. doi:10.1093/ageing/afm019 17374600

[6] Johnson EL, Sullivan KJ, Schneider ALC, et al. Association of plasma Abeta(42)/Abeta(40) ratio and late-onset epilepsy: results from the atherosclerosis risk in communities study. Neurology. 2023;101(13):e1319–e1327. doi:10.1212/WNL.0000000000207635 37541842 PMC10558158

[7] Johnson EL, Krauss GL, Lee AK, et al. Association between midlife risk factors and late-onset epilepsy: results from the atherosclerosis risk in communities study. JAMA Neurol. 2018;75(11):1375-1382. doi:10.1001/jamaneurol.2018.1935 30039175 PMC6248112

[8] Bell JS, Lonnroos E, Koivisto AM, et al. Use of antiepileptic drugs among community-dwelling persons with Alzheimer's disease in Finland. J Alzheimers Dis. 2011;26(2):231-237. doi:10.3233/JAD-2011-110200 21593561

[9] Winter JD, Kerns JW, Qato DM, et al. A national survey of nursing home clinicians to explain increased valproate prescribing. Am J Geriatr Psychiatry. 2025;33(11):1197-1206. doi:10.1016/j.jagp.2025.07.008 40846597 PMC12406165

[10] Candon M, Strominger J, Gerlach LB, Maust DT. Antiepileptic prescribing to persons living with dementia residing in nursing homes: a tale of two indications. J Am Geriatr Soc. 2023;71(1):89-97. doi:10.1111/jgs.18119 36349528 PMC9870979

[11] Ura H, Matsuoka N, Kubota K, Sadamoto K. Trends in prescription of anti-seizure medications in Japan between 2018 and 2021: a retrospective study using the National Database Open Data Japan. Epilepsy Behav. 2024;159:109949. doi:10.1016/j.yebeh.2024.109949 39121754

[12] Zelano J, Idegard A, Larsson D. Trends in epilepsy mortality of three incidence cohorts across 2006-2023 in Sweden: a matched register-based study. Lancet Reg Health Eur. 2025;56:101388. doi:10.1016/j.lanepe.2025.101388 40747516 PMC12310390

[13] Ludvigsson JF, Appelros P, Askling J, et al. Adaptation of the Charlson comorbidity index for register-based research in Sweden. Clin Epidemiol. 2021;13:21-41. doi:10.2147/CLEP.S282475 33469380 PMC7812935

[14] Nagga K, Bransvik V, Stomrud E, et al. Prevalence and ascertainment of dementia cases in the malmo diet and cancer study. J Alzheimers Dis Rep. 2022;6(1):529-538. doi:10.3233/ADR-220003 36275419 PMC9535608

[15] Bruun E, Virta LJ, Kalviainen R, Keranen T. Choice of the first anti-epileptic drug in elderly patients with newly diagnosed epilepsy: a Finnish retrospective study. Seizure. 2015;31:27-32. doi:10.1016/j.seizure.2015.06.016 26362374

[16] Mintzer S, Trinka E, Kraemer G, Chervoneva I, Werhahn KJ. Impact of carbamazepine, lamotrigine, and levetiracetam on vascular risk markers and lipid-lowering agents in the elderly. Epilepsia. 2018;59(10):1899-1907. doi:10.1111/epi.14554 30178473

[17] Chochol P, Arturo N, Lajczak PM, Rizwan Ahmed A, Koppanatham A, Varkey TC. Effects of antiseizure medications on lipid profile and weight in patients with epilepsy: a systematic review with meta-analysis. CNS Drugs. 2025;39(12):1221-1239. doi:10.1007/s40263-025-01231-2 41032224 PMC12602656

[18] Olesen JB, Abildstrom SZ, Erdal J, et al. Effects of epilepsy and selected antiepileptic drugs on risk of myocardial infarction, stroke, and death in patients with or without previous stroke: a nationwide cohort study. Pharmacoepidemiol Drug Saf. 2011;20(9):964-971. doi:10.1002/pds.2186 21766386

[19] Taipale H, Gomm W, Broich K, et al. Use of antiepileptic drugs and dementia risk-an analysis of finnish health register and German health insurance data. J Am Geriatr Soc. 2018;66(6):1123-1129. doi:10.1111/jgs.15358 29566430

[20] Papazian O, Canizales E, Alfonso I, Archila R, Duchowny M, Aicardi J. Reversible dementia and apparent brain atrophy during valproate therapy. Ann Neurol. 1995;38(4):687-691. doi:10.1002/ana.410380423 7574471

[21] Helmstaedter C, Beghi E, Elger CE, et al. No proof of a causal relationship between antiepileptic drug treatment and incidence of dementia. Comment on: use of antiepileptic drugs and dementia risk-An analysis of Finnish health register and German health insurance data. Epilepsia. 2018;59(7):1303-1306. doi:10.1111/epi.14432 29806877

[22] Vossel K, Ranasinghe KG, Beagle AJ, et al. Effect of levetiracetam on cognition in patients with Alzheimer disease with and without epileptiform activity: a randomized clinical trial. JAMA Neurol. 2021;78(11):1345-1354. doi:10.1001/jamaneurol.2021.3310 34570177 PMC8477304

[23] Cumbo E, Ligori LD. Levetiracetam, lamotrigine, and phenobarbital in patients with epileptic seizures and Alzheimer's disease. Epilepsy Behav. 2010;17(4):461-466. doi:10.1016/j.yebeh.2010.01.015 20188634

[24] French JA, Perucca E, Sander JW, et al. FDA safety warning on the cardiac effects of lamotrigine: an advisory from the Ad Hoc ILAE/AES Task Force. Epilepsia Open. 2021;6(1):45-48. doi:10.1002/epi4.12475 33681647 PMC7918301

[25] Everhov AH, Frisell T, Osooli M, et al. Diagnostic accuracy in the Swedish national patient register: a review including diagnoses in the outpatient register. Eur J Epidemiol. 2025;40(3):359-369. doi:10.1007/s10654-025-01221-0 40140143 PMC12137447

[26] Sveinsson O, Andersson T, Carlsson S, Tomson T. The incidence of SUDEP: a nationwide population-based cohort study. Neurology. 2017;89(2):170-177. doi:10.1212/WNL.0000000000004094 28592455

[27] Mbizvo GK, Bennett KH, Schnier C, Simpson CR, Duncan SE, Chin RFM. The accuracy of using administrative healthcare data to identify epilepsy cases: a systematic review of validation studies. Epilepsia. 2020;61(7):1319-1335. doi:10.1111/epi.16547 32474909

